# Immunogenic Human Papillomavirus Pseudovirus-Mediated Suicide-Gene Therapy for Bladder Cancer

**DOI:** 10.3390/ijms17071125

**Published:** 2016-07-14

**Authors:** Rim Hojeij, Sonia Domingos-Pereira, Marianne Nkosi, Dalila Gharbi, Laurent Derré, John T. Schiller, Patrice Jichlinski, Denise Nardelli-Haefliger

**Affiliations:** 1 Department of Urology, Centre Hospitalier Universitaire Vaudois (CHUV) and University of Lausanne, Lausanne 1011, Switzerland; reem.hojeij@gmail.com (R.H.); Sonia.domingos-pereira@chuv.ch (S.D.-P.); marianne.nkosi@epfl.ch (M.N.); dalilagh8@gmail.com (D.G.); laurent.derre@chuv.ch (L.D.); patrice.jichlinski@chuv.ch (P.J.); 2 Laboratory of Cellular Oncology, National Cancer Institute, National Institutes of Health, Bethesda, MD 20892, USA; schillej@dc37a.nci.nih.gov

**Keywords:** human papillomavirus vectors, tumor-targeting, immunogenic suicide-gene therapy, bladder cancer

## Abstract

Bladder cancer is the second most common urological malignancy in the world. In 70% of cases it is initially diagnosed as non-muscle-invasive bladder cancer (NMIBC) and it is amenable to local treatments, with intravesical (IVES) Bacillus-Calmette-Guerin (BCG) immunotherapy being routinely used after transurethral resection of the lesion. However, this treatment is associated with significant side-effects and treatment failures, highlighting the necessity of novel strategies. One potent approach is the suicide-gene mediated therapy/prodrug combination, provided tumor-specificity can be ensured and anti-tumor immune responses induced. Using the mouse syngeneic orthotopic MB49-bladder tumor model, here we show that IVES human papillomavirus non-replicative pseudovirions (PsV) can pseudoinfect tumors with a ten-fold higher efficacy than normal bladders. In addition, PsV carrying the suicide-gene herpes-simplex virus thymidine kinase (PsV-TK) combined to Ganciclovir (GCV) led to immunogenic cell-death of tumor cells in vitro and to MB49-specific CD8 T-cells in vivo. This was associated with reduction in bladder-tumor growth and increased mice survival. Altogether, our data show that IVES PsV-TK/GCV may be a promising alternative or combinatory treatment for NMIBC.

## 1. Introduction

Bladder cancer is the fourth and eighth most common malignancy among men and women, respectively [1,2]. Seventy percent of tumors present as non-muscle-invasive bladder cancer (NMIBC) at initial diagnosis with variable risk of recurrence and progression to invasive disease, thus requiring long-term surveillance [3]. For almost forty years, the gold-standard treatment has been intravesical (IVES) Bacillus-Calmette-Guerin (BCG) immunotherapy after transurethral resection of the lesion [4]. However, this treatment only partially limits tumor recurrence/progression [5], rendering these patients in need of new therapies. An attractive approach may be to initiate tumor self-destruction in an immunogenic way, such that tumor-antigen cross-presentation can be promoted and a tumor-specific adaptive immune response induced [6]. This goal might be achieved by intra-tumoral delivery and expression of a “suicide-gene”, which encodes an enzyme that catalyzes the formation of highly toxic metabolites when administered with a non-toxic prodrug. The best-known example is the herpes simplex virus thymidine kinase (HSV-TK) and Ganciclovir (GCV) combination [7]. HSV-TK is able to specifically phosphorylate the nucleoside analog GCV to GCV-monophosphate which is further converted by host kinases to GCV-triphosphates. The latter, when incorporated by polymerases during DNA synthesis, will act as chain terminator, thus perturbing replication and inducing tumor-cell killing [8]. To be immunogenic and lead to efficient anti-tumor responses, tumor cell-death (TCD) should result in the emission of danger signals that will trigger phagocytosis of cell debris and maturation of dendritic cells. At the molecular level, immunogenic TCD is characterized by the early surface exposure of Calreticulin (CRT), subsequent secretion of ATP, and release of High-Mobility Group Box-1 (HMGB-1) [9].

For safety reasons, a critical issue is the use of an effective and tumor-specific delivery system, a characteristic that may be provided by human papillomavirus (HPV)-based gene transfer vectors. HPV has a specific tropism for infecting the basal cells of stratified epithelia with binding to heparan sulfate proteoglycans (HSPG) on the basement membrane being an obligatory initial step in HPV infection in vivo [10]. The mechanisms of HPV infection have been mainly unraveled thanks to technologies enabling efficient packaging of DNA plasmids into HPV capsid proteins and generation of high titers of non-replicative pseudovirions (PsV) [11], which have shown efficacy in DNA delivery [12,13,14]. Interestingly, the specific subset of HSPG patterns of *N*- and *O*-sulfation to which HPV PsV bind [15] are not only enriched on the basement membrane, but also on the cell surface of immortalized cells in culture, tumor cells in vivo and in the extracellular matrix within the tumor microenvironment [16]. Accordingly, specific targeting to a variety of tumor cells-lines and solid tumors after intravenous (iv) or intraperitoneal (ip) delivery have been recently reported [17]. Here, we therefore tested the cancer-targeting abilities of HPV PsV after IVES administration towards implementation of a suicide-gene therapy for NMIBC. For this study, we used a mouse orthotopic model, where syngeneic bladder tumor cells (MB49, derived from a chemically induced urothelial carcinoma) are IVES instilled so that tumor deposition and seeding onto the mouse urothelium closely reproduces NMIBC [18]. Our data report that PsV-mediated gene therapy induces immunogenic TCD not only in vitro, but also in vivo, leading to induction of tumor-specific adaptive immune responses and increased mice survival.

## 2. Results

### 2.1. In Vitro Induction of Immunogenic Tumor Cell-Death (TCD) by PsV Encoding for Thymidine Kinase (PsV-TK)/Ganciclovir (GCV)

**Figure 1:**
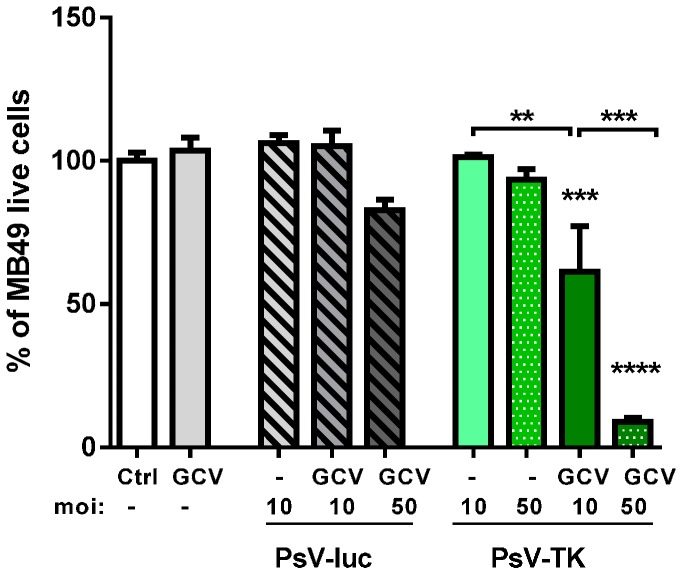
In vitro toxicity of pseudovirions encoding for thymidine kinase (PsV-TK)/Ganciclovir (GCV): Mean ± standard error of the mean (SEM) percentage (%) of viable MB49-cells 6 days after infection with PsV encoding for luciferase (PSV-luc) (stripped bars) or PsV-TK (green bars) at multiplicity of infection (moi) 10 or 50 and in presence or absence of GCV treatment (as indicated) or with GCV treatment alone (plain grey bar) as normalized to untreated cells (Ctrl, white bar). Significant differences with the control group following one-way ANOVA and Tukey post-test are indicated above the bars. Significant differences between specific groups are also shown: ** for *p <* 0.01, *** for *p <* 0.001 and **** for *p <* 0.0001.

The cytotoxic effect of pseudovirions encoding for thymidine kinase (PsV-TK) in combination with GCV was first examined in vitro on MB49-cells. PsV-TK or an irrelevant PsV encoding for luciferase (PsV-luc) were added to MB49-cells at a multiplicity of infection (moi) of 10 or 50, followed by addition or not, of GCV 24 h later. Five days later, viable cells were directly quantified using a one-solution colorimetric assay. The data show that only cells infected with PsV-TK and incubated with GCV presented a significantly lower viability, representing less than 10% of control cells when the higher moi of 50 was used (Figure 1).

**Figure 2:**
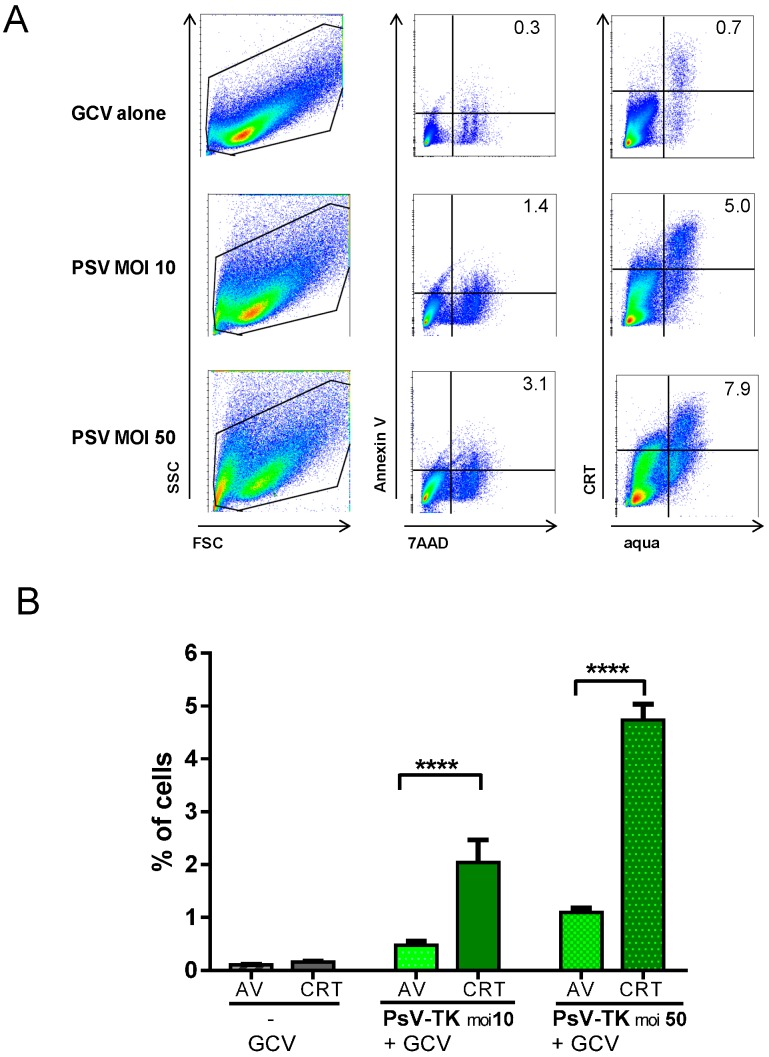
PsV-TK/GCV induced immunogenic cell-death in vitro: Flow cytometry analysis of MB49-cells recovered 3–4 days after infection with PsV-TK at moi 10 or 50 followed by GCV or treated with GCV alone and stained for AnnexinV (AV), Calreticuline (CRT) and dead/live markers (7AAD and aqua). A representative plot showing Forward-scattered light (FSC)/side-scattered light (SSC) (**left** plots), apoptosis (AV, 7AAD, **middle** plots) and CRT exposure (**right** plots) for each treatment is shown for each group in (**A**) Mean ± SEM percentage (%) of cells in early apoptosis (AV: AV^+^7AAD^−^) or with early CRT exposure (CRT: CRT^+^aqua^−^) are shown for each treatment groups (**B**). Significant differences between AV and CRT among each treatment group are shown following one-way ANOVA and Tukey post-test: **** for *p <* 0.0001.

TCD and its immunogenic potential were further evaluated by flow cytometry. MB49-cells infected with PsV-TK at moi 10 or 50 followed by GCV 24 h later were compared to treatment with GCV alone. Two days later, AnnexinV/7AAD staining was used to determine cells in early apoptosis (AnnexinV^+^7AAD^−^ cells, Figure 2A), while immunogenic TCD was evidenced by the early exposure of CRT (CRT^+^aqua^−^ cells, Figure 2A). It is noteworthy that, PsV-TK/GCV treatment induced significantly more cells with early CRT expression as compared to cells in early apoptosis (Figure 2B), confirming CRT exposure occurred upstream of apoptosis or necrosis, as part of a specific danger-signaling system [19]. In agreement with an immunogenic TCD, a high secretion of HMGB-1 was measured in the supernatant of cells after PsV-TK/GCV treatment (Figure S1). Altogether, the data shows that PsV-TK/GCV mediated suicide gene therapy of MB49-cells in vitro is effective and potentially immunogenic.

### 2.2. Preferential Targeting of MB49 Bladder-Tumors by PsV

**Figure 3:**
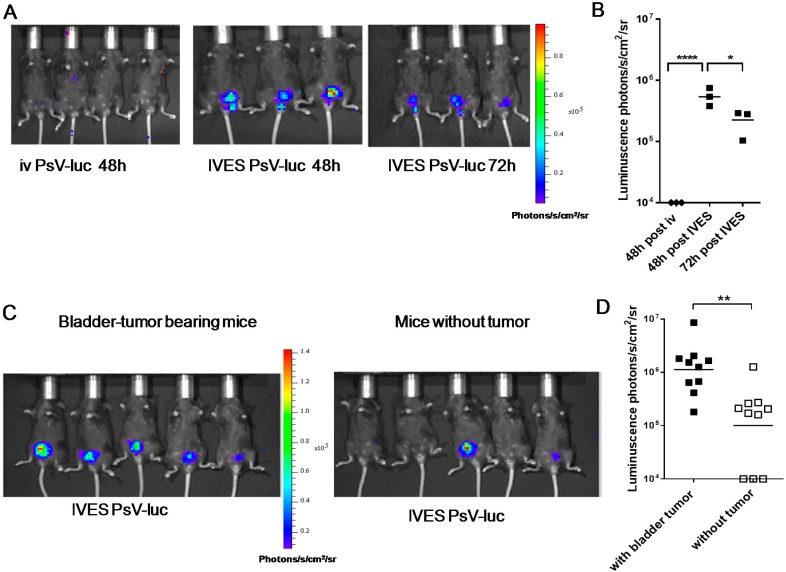
Bladder-tumor infection with PsV-luc: Efficacy of PsV-luc pseudoinfection was assessed by bioluminescence imaging 48 and/or 72 h after intravenous (iv) or intravesical (IVES) administration in mice bearing MB49 bladder-tumors (**A**,**B**) or 48 h after IVES administration in mice with or without bladder tumors (**C**,**D**). Representative Xenogen photographic images are shown in (**A**,**C**), while luminescence quantification of bladder/bladder tumors are shown in (**B**,**D**). Significant differences following a Student *t*-test are indicated by * for *p <* 0.05, ** for *p <* 0.01, and **** for *p <* 0.0001.

PsV-luc was used to visualize PsV pseudoinfection of the bladder by in vivo bioluminescence imaging after luciferin injection. IVES administration without prior treatment was ineffective (Figure S2). However, a five minute pre-treatment with a mild detergent (0.5% Nonoxynol-9, N9) was sufficient to allow PsV infection as visualized by luciferase expression in the bladder-tumor after 48 h and, though slightly decreased, at 72 h (Figure 3A,B). In contrast, iv administration of the same PsV-luc dose (10^6^ transducing relative light units transducing relative light units (TRLU) [20]) did not induce detectable expression in the mouse (Figure 3A,B). To assess tumor-targeting specificity we then compared infectivity/expression in mice bearing or not bladder tumors. The data showed a significantly 10-fold higher PsV-luc pseudoinfection of bladder tumors than of healthy bladder, in agreement with preferential infection of the tumors (Figure 3C,D).

### 2.3. PsV-TK/GCV Therapy Reduced Bladder-Tumor Growth and Increased Tumor-Specific CD8 T Cells and Mice Survival

**Figure 4:**
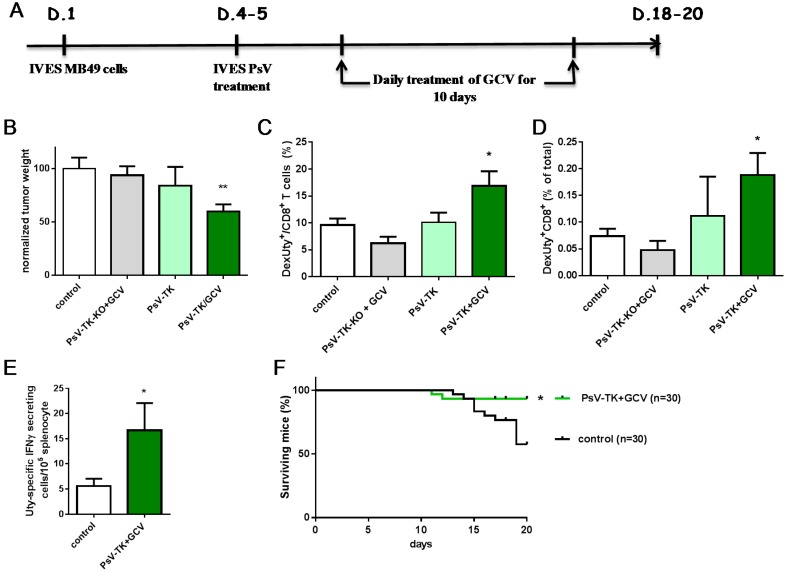
IVES PsV-TK/GCV increase tumor-specific CD8 T cells, tumor regression and mice survival: Tumor-bearing mice received different IVES PsV with or without ip GCV according to the time-line in days (D.) drawn in (**A**); Mean ± SEM normalized bladder-tumor weights (in relative units, RU) of mice that were alive at day 18–20, including untreated control mice (*n =* 18), mice that received the non-functional PsV-TK-KO + GCV (*n =* 4, grey bar), PsV-TK without GCV (*n =* 5, light green bar) and PsV-TK + GCV (*n =* 24) are shown in (**B**); Flow cytometry analysis of bladder tumors, for the presence of MB49-specific CD8 T-cells (DexUty^+^CD8^+^) is shown as percentage (mean ± SEM) among CD8 T-cells in (**C**) and among total cells in (**D**); Spleen of control mice (*n =* 21) and PsV-TK + GCV treated mice (*n* = 17) were analyzed by ELISPOT for the presence of effector MB49-specific CD8 T-cells (Uty-specific IFN-γ secreting cells), mean ± SEM/10^5^ cells are shown in (**E**); Surviving mice over time are shown for the PsV-TK + GCV treatment (green line) and control mice (black line) in (**F**). Significant differences following one-way ANOVA and Tukey post-test for **B**, **C** and **D**, a student *t*-test for **E** and a log-rank test for **F** are indicated by * for *p <* 0.05, ** for *p <* 0.01.

PsV-TK/GCV suicide gene therapy was evaluated in vivo after IVES instillation of 10^6^ transducing relative units (TRU [21]) of PsV-TK in mice harboring day 4–5 MB49 bladder-tumors, followed 2 days later by a 10-days long daily ip GCV treatment as indicated in Figure 4A. Mice receiving PsV-TK-KO (a PsV expressing a non-functional TK protein [22]) and GCV or mice receiving PsV-TK without GCV were also used for comparing to untreated control mice. Surviving mice were killed at days 18–20 and their bladder, which included the tumor, was weighted (Figure 4B). The data shows that only the mean bladder-tumor weight of mice receiving PsV-TK/GCV treatment was significantly lower than control mice. To examine whether the adaptive tumor-specific immune responses may be modulated by the PsV-TK/GCV suicide gene therapy in vivo, an antigen specifically expressed by MB49 tumor cells, the minor histocompatibility male antigen HY (Uty) [23] and the known H-2D^b^ epitope Uty_246–254_ [24] were used to measure the anti-tumor CD8 T-cell responses. Bladder-tumors were recovered and stained for the presence of intratumoral Dextramer Uty-specific CD8 T-cells (Figure 4C,D). Flow cytometry analysis shows that Uty-specific CD8 T-cells were significantly increased by the PsV-TK/GCV treatment as compared to control mice. In addition, analysis of splenocytes by IFN-γ ELISPOT showed a significantly higher proportion of effector Uty-specific CD8 T-cells upon PsV-TK/GCV therapy (Figure 4E). This suggests that the immunogenic tumor-killing induced by PsV-TK/GCV has resulted in tumor-antigen presentation and MB49-specific CD8 T-cell induction. Finally, survival of PsV-TK/GCV treated mice (93%) was significantly improved as compared to control mice (58%, Figure 4F). Altogether, the data show that PsV-TK/GCV treatment is immunogenic and effective in vivo, at least at short term.

## 3. Discussion

In this study, we showed that HPV PsV can successfully and preferentially pseudoinfect bladder tumors after IVES administration in mice, resulting in immunogenic PsV-TK/GCV-mediated TCD with induction of tumor-specific CD8 T-cells, decreased bladder tumor-weight and increased mice survival.

Efficacy and safety of suicide-gene therapy is primarily linked to the delivery vector used, the ideal being high efficiency-delivery to tumor-cells while not to other dividing normal cells. Specific and efficient delivery of HPV PsV or virus-like particles to ovarian or lung tumors in mouse orthotopic models was recently reported after either ip or iv administration [17,25]. NMIBC are superficial tumors which are lining the urothelium, confined within the bladder mucosa, and thus more easily reached through the IVES route. However, a glycosaminoglycan layer acts as a natural protective barrier not only to external substances, but also to tumor implantation in the mice, which then requires some prior mechanical or chemical disruption of the urothelium [26]. This may lead to implantation of tumors which are surrounded by a regenerating “normal” urothelium, which will then impede direct access of HPV PsV to HSPG on the tumor. To overcome this problem, we have used a very mild treatment with N9, a known epithelial disrupter [27], so that a 10-fold preferential infection of the bladder-tumor, as compared to healthy bladder, could be demonstrated. Prior surface disruption, and thus risk of exposing healthy basal membrane associated with a decrease in PsV tumor-targeting abilities, will likely not be necessary for effective treatment of incipient NMIBC lesions in patients. Indeed, the tumoral urothelium is readily accessible as shown by histological studies [28], efficacy of in vivo Hexaminolevulinate fluorescence cystoscopy for carcinoma in situ diagnostic [29] or of IVES BCG immunotherapy [30]. Obviously, it will be important to determine whether preferential targeting of HPV PsV for the tumoral urothelium can be confirmed in NMIBC patients.

HSV-TK/GCV suicide-gene therapy has been extensively investigated in the past [8,31], with previous report of immunogenic TCD [32]. These studies have included bladder cancer murine models (reviewed in [33]), though induction of tumor-specific immune responses were not previously investigated. Our data show, with pre-apoptotic CRT exposure and HMGB-1 release, the immunogenic nature of PsV-TK/GCV-mediated MB49 TCD in vitro, and more importantly its translation into increased MB49-specific CD8 T-cells numbers in vivo. This resulted in smaller bladder-tumors at day 20, similar to data obtained in an ovarian tumor model [25], but also an improved mice survival. Although these data are promising, we anticipate that multiple or combinatory therapies will be necessary to ensure long term mice survival, as already observed with other suicide-gene treatments where, when combined with immunostimulation and/or tumor vaccination, these treatments were able to enhance immune-mediated killing [34,35,36] and long term tumor protection [37]. One advantage of HPV-vectors is that their administration results in capsid-neutralizing antibodies which are HPV-type specific and, thanks to the availability of a high variety of HPV-types, multiple successive treatments can be easily envisioned [13].

## 4. Materials and Methods

### 4.1. MB49 Cells and the Orthotopic Bladder Tumor Model

The MB49 cell-line (kindly provided by Prof. Angelica Loskog, Uppsala University, Sweden) is derived from a carcinogen induced urothelial carcinoma in male C57Bl/6 mice [18]. Seven to ten-week-old female C57Bl/6 wild type mice (Charles River, Lyon, France) were used in compliance with ethical directives of the Swiss veterinary authorities. Bladder-tumors were established in deeply anesthetized mice that were IVES catheterized using Introcan 24Gx¾” catheters (B. Braun, Melsungen, Germany). A 15 min pre-treatment with 100 µL 22% ethanol was performed before instillation of 200,000 MB49-cells in 50 µL. Mice were carefully monitored for health status and hematuria and were euthanized in case of >15% weight-loss.

### 4.2. PsV Production and PsV/GCV Suicide Gene Therapy in Vitro and in Vivo

PsV-TK, PsV-TK-KO and PsV-luc were produced according to Buck et al. [11] using the p16L1h and p16L2h plasmids [38]), encoding for the HPV16 capsid, and the corresponding encapsidated plasmids (phTKf, phTK-KOf or pCLucf). Briefly PsV were purified on Optiprep Gradient by ultracentrifugation from 293TT cells [11] transfected with the selected plasmids 3 days earlier. PsV-TK, PsV-TK-KO TRU and PsV-luc TRLU titers were determined as previously described [20,21]. For in vitro PsV/GCV mediated killing, different moi of PsV were added to MB49 cells for 24 h and 100 µM GCV (InvivoGen, Toulouse, France) was then added for 2 to 5 days. Viable cells were measured using the CellTiter 96^®^ Aqueous One Solution Cell Proliferation assay kit (Promega, Madison, WI, USA) according to manufacture instructions. For determination of AnnexinV and CRT expression by flow cytometry, cells were stained with live-dead marker aqua (from Invitrogen life technologies, Carlsbad, CA, USA) and 7AAD (from the apoptosis detection kit I from BD Pharmingen, San Diego, CA, USA), AnnexinV-PE (from the same apoptosis detection kit), rabbit anti-mouse CRT antibody (ab2907, Abcam, Cambridge, UK) and anti-rabbit IgG F(ab’)_2_ APC-Cy7 secondary antibody (Santa Cruz biotechnology, Dallas, TX, USA). Cell acquisition and analysis were performed using Gallios Flow Cytometer (Beckman Coulter, Nyon, Switzerland) and FlowJo software (Tree Star Inc., Ashland, OR, USA), respectively. HMGB-1 content in the supernatant of the treated cells was determined by an ELISA according to manufacturer instructions (IBL International, Hamburg, Germany). For in vivo PsV/GCV treatment, deeply anesthetized mice, catheterized as described above, received 0.5% Nonoxynol-9 (Igepal, Sigma, St. Louis, MO, USA) for 5 min and, after a thorough phosphate-buffered saline rinsing of the bladder, were instilled with 10^6^ TRU of PsV. Two days later, mice received daily intraperitoneal (ip) injections of GCV (75 µg/g of body weight) for 10 days.

After IVES instillation of PsV-luc, luciferase expression was monitored by bioluminescence 15 min after ip injection of d-luciferin (150 μg/g of body weight, Promega, Dübendorf, Switzerland) in the Xenogen imaging system (Xenogen/IVIS Caliper Life Science, kindly provided by cellular imaging facility, CIF/UNIL, Lausanne, Switzerland).

### 4.3. Preparation of Murine Cells, IFN-γ ELISPOT and T-Cell Labeling

Single-cell suspensions from the bladders and spleens of sacrificed mice were obtained as previously described [39,40]. Briefly, splenocytes were obtained by mechanical dissociation, while bladders were minced and digested step-wise with 0.5 mg/mL thermolysin (Roche, Basel, Switzerland) and 1 mg/mL collagenase/dispase (Roche). IFN-γ ELISPOT assays were performed as described [39] using Multiscreen-HA 96-well plates (MAHA S4510, Millipore, Billerica, MA, USA), anti-IFN-γ monoclonal antibody (R4-6A2, Beckton Dickinson Pharmingen), biotinylated anti-IFN-γ monoclonal antibody (XMG1.2, Beckton Dickinson Pharmingen) and Streptavidin-AP (Roche). 100,000 splenocytes/well in duplicates were incubated with 0.5 μg/mL of H-2D^b^ restricted Uty_246–254_ peptide or medium alone (control wells) for 16–24 h. Uty-specific responses were defined as the number of IFN-γ spots/10^5^ cells in the Uty-stimulated wells minus the number of IFN-γ spots/10^5^ cells in the control wells (<3 spots/well). T cell staining was performed as previously described [41], using PE-conjugated Uty_246–254_ H-2D^b^-restricted dextramers (Immudex, København, Denmark) and APC-labeled CD8α (clone 53-6.7, eBioscience, San Diego, CA, USA). Flow-cytometer cell acquisition and analysis were performed as described above.

### 4.4. Statistical Analysis

Statistical analyses were performed using Prism 7.00 for Windows (GraphPad software, La Jolla, CA, USA). Multiple comparisons were performed using one-way ANOVA and Tukey’s Multiple Comparison Test, Student *t*-test or log-rank test as indicated in the figure legends.

## 5. Conclusions

The bladder is an ideal organ for evaluating novel in situ therapies, because IVES instillation can maximize exposure to the local tumor, while minimizing systemic exposure and limiting toxicity [42]. Bladder cancer has a high prevalence, a poor prognosis if diagnosed late or treated inadequately, and a high socio-economic cost [43]. The most prevalent form of the disease, NMIBC makes a large contribution to this figure and no major progress has been made in the last twenty years in patient treatment. HPV-vector-mediated tumor-targeting and improved immunogenic killing may be an innovative therapy after transurethral resection of NMIBC and possibly could be applied to other types of cancers amenable to topical treatment.

## Supplementary Materials

Supplementary materials can be found at http://www.mdpi.com/1422-0067/17/7/1125/s1.

## Abbreviations

BCG	Bacillus-Calmette Guérin
CRT	Calreticuline
GCV	Ganciclovir
HSPG	Heparan sulfate proteoglycans
HSV	Herpes simplex virus
HMGB-1	High-mobility group box-1
HPV	Human papillomavirus
IVES	intravesical
NMIBC	Non muscle-invasive bladder cancer
PsV	Pseudovirion
TCD	Tumor cell-death
TK	Thymidine kinase
TK-KO	Non functional TK
TRLU	transducing relative light units
