# Supplementary material for: Immunogenic Human Papillomavirus Pseudovirus-Mediated Suicide-Gene Therapy for Bladder Cancer
Source: Int J Mol Sci. 2016 Jul 14;17(7):1125. doi: 10.3390/ijms17071125 (PMC4964499; doi:10.3390/ijms17071125)
Supplement: Supplementary file 1 [file ijms-17-01125-s001.pdf]

Supplementary Materials: Immunogenic Human Papillomavirus Pseudovirus-Mediated Suicide-Gene Therapy for Bladder Cancer

Rim Hojeij, Sonia Domingos-Pereira, Marianne Nkosi, Dalila Gharbi, Laurent Derré, John T. Schiller, Patrice Jichlinski and Denise Nardelli-Haefliger

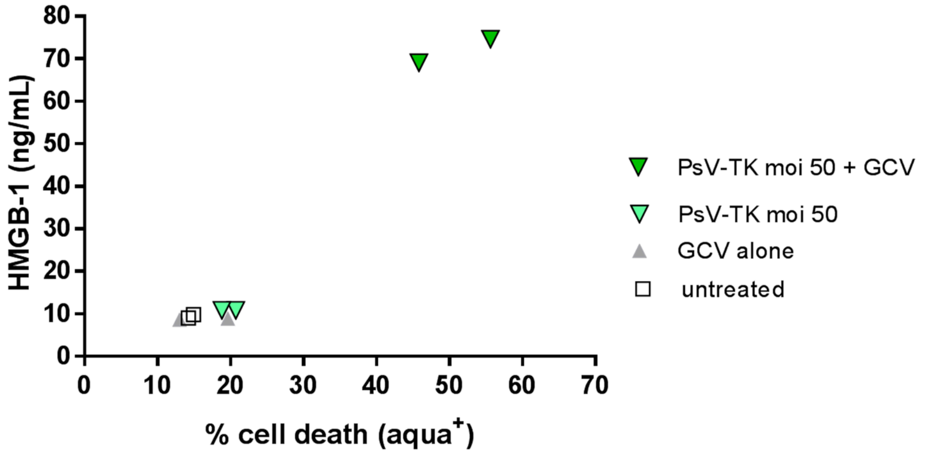

Figure S1. High-Mobility Group Box-1 (HMGB-1) secretion upon pseudovirions encoding for thymidine kinase (PsV-TK)/Ganciclovir (GCV) killing of MB49 cells in vitro: HMGB-1 (ng/mL) was measured by ELISA in the supernatant of MB49-cells infected 5 days earlier with PsV-TK at multiplicity of infection (moi) 50 with (dark green triangle) or without GCV (light-green triangle), or receiving GCV alone (grey triangle) or left untreated (white square). Results were plotted against the respective percentage (%) of cell death induced as measured by flow cytometry (aqua+ cells).

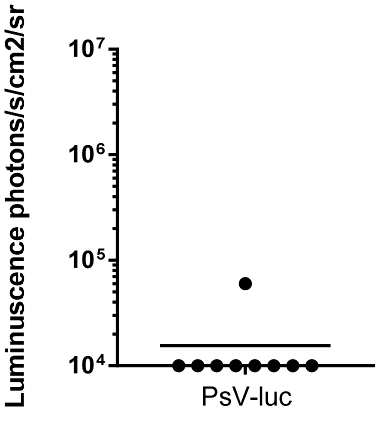

Figure S2. Intravesical (IVES) instillation of Pseudovirions encoding for luciferase (PsV-luc) is inefficient in absence of N9 prior treatment. Day 5 MB49-bladder tumor bearing mice received IVES instillation of 10^6 transducing relative light units (TRLU) of PsV-luc without any pretreatment. Quantification of bladder/ tumor luminescence 48 h.
